# *Lelliottia wanjuensis* sp. nov. Isolated from Korean Lettuce in Wanju, South Korea

**DOI:** 10.1007/s00284-024-03911-6

**Published:** 2024-09-28

**Authors:** Jiyeon Lee, Eewon Tai, Sunyoung Jeong, Ile Kim, Bo-Eun Kim, Myeong-In Jeong, Kwang-Kyo Oh, Natalia Biere, Charles M. A. P. Franz, Gyu-Sung Cho

**Affiliations:** 1 Department of Microbiology and Biotechnology, Max Rubner-Institut, Federal Research Institute of Nutrition and Food, Hermann-Weigmann-Straße 1, 24103 Kiel, Germany; 2 Microbial Safety Division, National Institute of Agricultural Sciences, Rural Development Administration, Wanju, 55365 Republic of Korea

## Abstract

Ten Gram stain-negative, oxidase-negative, catalase-positive, rod-shaped strains were isolated from lettuce in the city Wanju in South Korea. Comparative 16S rRNA gene analysis and multilocus sequence analysis indicated that the strains grouped closely together with all other *Lelliottia* (*L*.)-type strains. The average nucleotide identity (ANI) comparisons of the isolates showed a high relationship between the strains, as the ANI values of all strains ranged from 91.01 to 91.31% when compared to the *L. jeotgali-*type strain PFL01^T^. All isolates showed the highest genomic DNA sequence similarity to the *L. jeotgali* PFL01^T ^strain at 43.3% when compared with digital DNA–DNA hybridization (dDDH). Strain V104_15^T ^of the proposed novel *Lelliottia* species showed 91.2% and 43.3% similarity with the most closely related *L. jeotgali* PFL01^T^-type strain in the ANI and dDDH comparisons, respectively. Strain V104_15^T^ could not grow at 45 °C and 7% NaCl, while *L. jeotgali* PFL01^T^ strain could grow at both these conditions. Strain V104_15^T^ showed *ß*-glucosidase activity but not α-glucosidase activity, while the *L. jeotgali* PFL01^T^ strain was *α*-glucosidase positive but ß-glucosidase negative. The major cellular fatty acids of strain V104_15^T^ were C_16:0_ and cyclo-C_17:0_ including summed features. The mol % G + C content of the genomic DNA of strain V104_15^T^ was 55.74%. Phenotypic and biochemical characteristics, as well as the phylogenomic analysis indicated that the strain V104_15^T^ represents a novel species of the genus *Lelliottia*, for which the name *Lelliottia wanjuensis* sp. nov. is proposed. The type strain is V104_15^T^ (= LMG 32996^ T^ = DSM 115585^ T^).

**Supplementary Information:**

The online version contains supplementary material available at 10.1007/s00284-024-03911-6.

## Introduction

Bacteria belonging to the genus *Lelliottia* are Gram-stain-negative rods, motile, facultative anaerobic, and non-spore forming. They have been isolated from various ecological origins, including foods (onion, raw milk, cream, cheese, Spanish pork sausage) as well as drinking water and trees [1–3]. Two species previously belonging to the *Enterobacter* genus, i.e., *Enterobacter amnigenus* and *Enterobacter nimipressuralis,* were reclassified into the novel genus *Lelliottia* as *Lelliottia* (*L.*) *amnigena* comb. nov. and *Lelliottia nimipressuralis* comb. nov., respectively [4]*.* In that previous study, the genotypic results of multilocus sequence analysis (MLSA) based on concatenated *gyr*B, *rpo*B, *inf*B, and *atp*D gene sequences and in silico DNA–DNA hybridization (dDDH), as well as data on phenotypic characteristics and fatty acid profiles in cell wall membranes, supported the genus separation from *Enterobacter* [4]. Currently, the *Lelliottia* genus comprises only three species, i.e., *L. jeotgali*, *L. nimipressuralis,* and *L. amnigena* [4, 5]. The previously described species *L. aquatilis* [6] was recently reclassified as a later heterotypic synonym of *L. jeotgali,* based on the results of the average nucleotide identity (ANI) and in silico DDH of the two species [7].

In recent years, the consumption of fresh produce and fruits has significantly increased and the potential infectious human pathogens (*Salmonella*, *Listeria monocytogenes*, and Shiga toxin-producing *Escherichia coli*), as well as opportunistic pathogens (*Klebsiella*, *Enterobacter*, and *Citrobacter*), have been detected in previous studies from fresh produce and fruits [8–10]. Furthermore, our previous study of Jeong et al. [11] highlighted the isolation of antibiotic-resistant Gram-stain-negative bacteria from lettuce and surrounding soil in Korea. In this study, ten isolates of a new species belonging to the *Lelliottia* genus are proposed based on phenotypical and genotypical, as well as whole-genome sequence analysis data. Thus, physiological and biochemical analyses, including a phylogenetic analysis using whole-genome sequence, clearly identified these ten strains isolated from lettuce in Korea as a novel species in the genus *Lelliottia*, for which the name *Lelliottia wanjuensis* is proposed*.*

## Materials and Methods

### Isolation and Cultivation

All strains were isolated from lettuce (*Lactuca sativa*) from test beds at the Korean National Institute of Agricultural Sciences (NAS) between June 29, 2021 and July 28, 2021 [11]. Briefly, twenty-five grams of outer leafy lettuce samples were gathered from the four distinct test beds in the city of Wanju, South Korea (35° 49′ 49.35″ N, 127° 3′ 29.91″ E). Each sample was placed in a stomacher bag (3 M, Seoul, Korea) with 225 mL of buffered peptone water (Difco, Sparks, MD, USA) and homogenized (Interscience, Saint-Nom la Breteche Arpents, France) for 2 min at maximum speed. For isolation of bile-tolerant Gram-stain-negative bacteria, 0.1-ml samples of appropriate dilutions prepared with quarter-strength Ringer’s solution (Merck, Darmstadt, Germany) were spread plated onto crystal violet neutral-red bile dextrose agar (VRBD) (Merck) plates. After incubation for 24 h at 37 °C, the well-separated colonies of different morphologies were randomly picked from each plate. Ten selected colonies were purified by successive rounds of growing in Luria Bertani (LB) broth (Carl Roth, Karlsruhe, Germany) and streaking out onto LB agar. All isolates were also routinely cultured in LB (Roth) broth at 35 °C for 18-h aerobic incubation. For long-term storage, the isolates were stored in this medium at − 80 °C with 25% (v/v) glycerol. The type strain candidate V104_15^T^ was deposited in the Deutsche Sammlung von Mikroorganismen und Zellkulturen culture collection (= DSM 115585^ T^) and in the Belgian coordinated culture collection (BCCM) of the Laboratory of Microbiology (= LMG 32996^ T^).

### Whole-Genome Sequence Analysis and Phylogeny

The total genomic DNA of all isolates was extracted using the peqGOLD bacterial DNA extraction kit (VWR, Darmstadt, Germany) following the manufacturer’s instructions. The concentration and quality of extracted DNA were measured using a Qubit 3 fluorometer (Invitrogen, Darmstadt, Germany) and NanoDrop spectrophotometer (Peqlab). The sequencing library preparations for both Illumina MiSeq and NextSeq sequencing were done following the manufacturer’s protocol. The 2 × 250 bp paired-end sequencing with Miseq (strains V86_10, V106_9, V106_10 and V106_16) and the 2 × 150 bp paired-end sequencing with NextSeq 500 (strains V89_13, V89_5, V89_10, V104_15^T^, V106_5, and V106_12) were performed according to the manufacturer’s instructions (Illumina, Munich, Germany). The barcodes and adapter sequences were trimmed from the raw sequences obtained from MiSeq and NextSeq using the Trimmomatic pipeline (v. 0.32; parameters: Phred 33, sliding window; 4:15, leading; 3, and minlen; 45) [12], and de novo genome assembly was performed using SPAdes (v.3.15.5.) [13] with parameter isolate. After genome assembly, the quality of the obtained contigs was determined with the QUAST tool (v. 5.0.2) [14] and all contigs shorter than 500 bp were removed using the bbmap pipeline (BBTools—DOE Joint Genome Institute). The PhiX-contaminated contigs were removed using the bbduk pipeline with default parameters (BBTools—DOE Joint Genome Institute). The obtained contigs were annotated using the Bacterial and Viral Bioinformatics Resource Center (BV-BRC) web-based tool [15, 16], Prokka (v. 1.14.0) [17], and the NCBI Prokaryotic Genome Annotation Pipeline version 4.13 [18]. Additionally, the acquired antibiotic resistance genes were identified using the ResFinder (database: 2023-04-12) [14, 15], and the plasmid replication-type sequences were identified using the PlasmidFinder pipeline (database: 2023-01-18) [16], respectively, with default parameters. Both analysis tools are available on the Center for Genomic Epidemiology (CGE) website.

To identify the isolated strains, the 16S rRNA gene sequences of each strain were extracted from the whole-genome sequence data and were compared for identification using the EzBioCloud database (https://www.ezbioclud.net/identify) [19]. Additionally, phylogenetic analysis based on 16S rRNA sequences was performed using the MEGA (v.11) [20] program using the maximum-likelihood method and Kimura two-parameter model. Furthermore, multilocus sequence analysis (MLSA) based on four housekeeping genes (ATP synthase beta subunit (*atpD*), DNA gyrase (*gyrB*), initiation translation factor 2 (*infB*), and RNA polymerase beta subunit (*rpoB*)) was conducted based on the previous study [21]. For this MLSA, the sequences of 4 housekeeping genes from these 10 isolates were extracted from whole-genome sequence data. These housekeeping genes were then concatenated into sequences of equal lengths and aligned with closely related type strains and other *Enterobacteriaceae* strains. Also, comparative phylogenetic tree analysis of MLSA sequences was performed with the MEGA (v.11) program [20] using the maximum-likelihood method and Kimura two-parameter model. Bootstrap values were calculated based on 1000 replications.

Additionally, whole-genome sequence-based phylogeny was performed using the BV-BRC server [16] with previously described *Lelliottia-*type strains and closely related *Enterobacteriaceae-*type strains. For this, 100 conserved coding sequences were extracted, concatenated, and filtered based on sequence homology. Subsequently, sequence alignment was performed using the MAFFT pipeline and a phylogenetic tree with 100 bootstrap values was estimated using the RAxML pipeline [16]. The ANI and the in silico DDH values were calculated using the OrthoANI (v. 0.93.1) [22] supported by BLASTn tool [23] and the Genome-to-Genome Distance Calculator (v. 3.0) with formula 2 [24], respectively.

## Phenotypic Characterization and Cell Wall Fatty Acid Analysis

The API 32E-miniaturized identification system was used for determining carbohydrate fermentation and enzyme activities. For this, bacteria were first sub-cultured in LB (Roth) agar plate for 18 h at 35 °C. A single colony was used to adjust the bacterial density to a McFarland density of 0.5 using 0.85% NaCl (w/v). This suspension was used to inoculate the API 32E test strips. The strips were incubated according to the manufacturer’s instructions. To determine salt tolerance, the strains were inoculated into LB broth containing NaCl (Roth) concentrations ranging from 0.5 to 10% and incubated at 35 °C for 120 h. The optimum growth temperature was determined by observing the cell growth after incubation at temperature ranging from 4 to 41 °C for 120 h. The cell wall fatty acid profile analysis of the novel species candidate V104_15^T^ was carried out commercially by the DSMZ services, Leibniz-Institut DSMZ (Deutsche Sammlung von Mikroorganismen und Zellkulturen) (Braunschweig, Germany). For cellular fatty acid analyses, cells were grown on LB agar at 35 °C for 18 h. The fatty acid methyl ester mixtures were separated by gas chromatography followed by instruction’s manual and were carried out using Sherlock Microbial Identification System (TSBA; version, 6.1 library). Peaks were automatically integrated and fatty acid names and percentages calculated by the MIS Standard Software (Microbial ID).

### Electron Microscopy

For transmission electron microscopy, 500 µl of fresh culture grown in LB (Roth) was centrifuged at 10,000 rpm for 1 min, the supernatant was discarded, and the pellet was re-suspended in 500 µl of Ringer’s solution (Merck). This process was repeated, with the second centrifugation performed for 2 min and the pellet was finally re-suspended in 200-µl Ringer’s solution. The sample was then fixed on carbon films using 1% glutaraldehyde for 10 min, followed by washing with distilled water. Negative staining was carried out using 2% uranyl acetate. The images were acquired with a Talos L120C (Thermo Fisher, Dreieich, Germany) electron microscope equipped with a CETA 16 M camera (Thermo Fisher) at an acceleration voltage of 80 kV.

## Results and Discussion

### Phenotypic and Biochemical Characteristics

An overview of the phenotypic and biochemical properties of the representative strain V104_15^T^ (= LMG 32996^ T^, = DSM 115585^ T^) of the novel species and other *Lelliottia* species is listed in Table 1. Additionally, the phenotypic characteristics of all non-type strain isolates belonging to the *L. wanjuensis* species were indicated in Suppl. Table 2. All ten isolates were Gram-stain-negative, rod-shaped bacteria with colony sizes ranging from 2.0 to 3.5 mm after overnight incubation at 35 °C on LB agar plate. The colonies were round to irregular, slightly convex, and slightly opaque. Growth occurred at temperatures of 7 °C to 39 °C and in the presence of 0.5–7% NaCl. Various phenotypic features obtained from the API 32E test system as shown in Table 2 for the type strain are also shown in Supplementary Table 2 for the other nine related *L. wanjuensis* strains isolated in this study. A distinctive characteristic of the species *L. wanjuensis* was that type strain candidate V104_15^T^ could not grow at 41 °C and 7% NaCl, while the closely related *L. jeotgali* PFL01^T^ strain could grow at both conditions. On the other hand, the strain V104_15^T^ exhibited ß-glucosidase activity but did not possess α-glucosidase activity, while *L. jeotgali* PFL01^T^ strain displayed α-glucosidase but not ß-glucosidase activity.

**Table 1:** Phenotypic characteristics of *L. wanjuensis* V104_15^T^ and the type strains of the species of the genus *Lelliottia*

Characteristics	V104_15^T^	*L. jeotgali* PFL01^T^	*L. nimipressuralis* CCUG 25894^ T^	*L. amnigena* LMG 2784^ T^
Growth at 41 °C	−	+	n.d	n.d
Growth at 7 °C	+	−	n.d	+
Growth at 6% (w/v) NaCl	+	+	n.d	n.d
Growth at 7% (w/v) NaCl	−	+	n.d	n.d
Enzyme activity:				
Urease	−	n.d	−	+
ß-glucosidase	+	−	+	+
α-glucosidase	−	+	+	+
α-galactosidase	+	+	n.d	n.d
Acid production from:				
D-trehalose	+	+	n.d	n.d
L-rhamnose	+	w	+	+
Inositol	−	−	−	−
D-cellobiose	+	w	+	+
D-sorbitol	−	−	+	−
5 ketogluconate	−	+	−	−
Indole (L-tryptophan)	w	n.d	−	−
D-sucrose	+	−	−	+

+: positive; w: weak positive; −: negative; n.d., not determined. Phenotypic data for *L. jeotgali* PFL01^T^, *L. nimipressuralis* CCUG 25894^T^ and *L. amnigena* LMG 2784^T^ were obtained from a previous study [5]

**Table 2:** Summary of whole-genome sequencing of *Lelliottia* strains isolated from lettuce

	No. of contigs	N_50_	G + C content (mol %)	Total length (bp)	Genome coverage	No. of CDSs	No. of tRNAs	No. of rRNAs^a^	Acquired resistance gene(s)^b^	^3^Plasmid sequence	GenBank accession no
V86_10	80	176,232	55.22	5,372,274	× 37	5146	78	15	*oqxB*	n.d	JASSOT010000000
V89_5	41	320,657	55.84	4,846,481	× 41	4569	62	8	*oqxA*, *oqxB*, *fosA*2	pKP1433	JASSOS010000000
V89_10	42	192,197	55.84	4,846,058	× 33	4559	56	7	*oqxA*, *oqxB*, *fosA*2	pKP1433	JASSOR010000000
V89_13^1^	94	115,470	55.84	4,828,316	× 85	4574	48	7	*oqxA*, *oqxB*, *fosA*2	pKP1433	JASCAP010000000
V104_15^T^	52	180,308	55.74	5,002,032	× 39	4691	60	8	*oqxB*, *fosA*2	n.d	JASSOQ010000000
V106_5	47	236,152	55.56	5,153,483	× 55	4841	77	20	*oqxB*, *fosA*	n.d	JASSOP010000000
V106_9	60	257,751	55.26	5,323,413	× 47	5067	75	15	*oqxB*	n.d	JASSOO010000000
V106_10	50	236,152	55.56	5,154,923	× 49	4846	77	19	*oqxB*, *fosA*	n.d	JASSON010000000
V106_12	157	73,097	55.24	5,240,512	× 66	5023	55	7	*oqxB*	n.d	JASSOM010000000
V106_16	52	319,671	55.63	5,155,048	× 48	4860	75	17	*oqxB*, *fosA*	n.d	JASSOL010000000

n.d., not detected. ^1^Whole-genome sequence data of V89_13 strain was adapted from a previous study [8]

^a^Number of RNAs are include partial rRNAs

^b^The acquired antibiotic resistance genes and plasmid sequence type were identified using the ResFinder (database: 2023-04-12) [14, 15] and the PlasmidFinder (database: 2023-01-18) [16], respectively, with default parameters

### Genomics and Phylogenomic Analysis

**Fig. 1:**
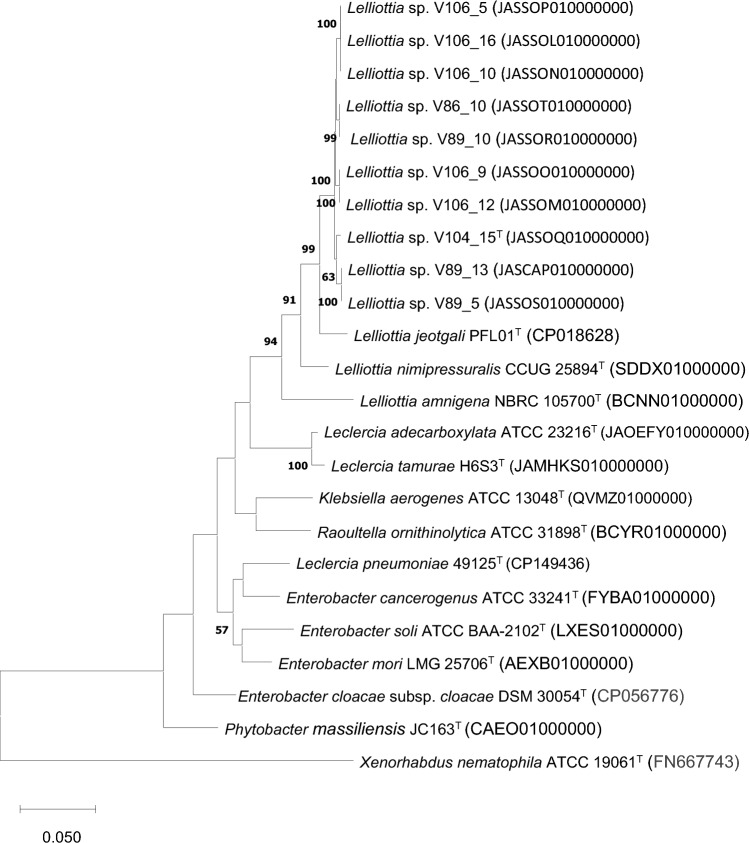
MLSA with closely related *Lelliottia*-type strains using the MEGA (v.11) program and the maximum-likelihood method and Kimura two-parameter model. Bootstrap values were calculated based on 1000 replications, with values over 50% being indicated. Bar indicates substitutions per nucleotide position

The genome sizes of all ten isolates were between 4.8 and 5.3 Mbp and their mol% G + C contents ranged from 55.22 to 55.84 mol%. The whole-genome sequences consisted of 41–157 contigs, with N_50_ values ranging from 73,097 to 320,657 (Table 2). The DDBJ/ENA/GenBank accession numbers for the genome sequences of all isolates are listed in Table 2. Phylogeny based on 16S rRNA gene sequences extracted from whole-genome sequence data, the strains were preliminary identified as *Lelliottia* species (Suppl. Figure 1). All ten strains isolated from lettuce showed high 16S rRNA gene sequence similarities to *Lelliottia-*type strains (over 99.4%), and the phylogenetic tree based on 16S rRNA gene sequences indicated that all isolates grouped closely together with the *Lelliottia-*type strains. Furthermore, MLSA-based phylogenetic analysis together with *Lelliottia*-type strains, including also closely related *Leclercia* and *Enterobacter-*type strains, clearly indicated that all lettuce isolates were distinct from the most related other *Lelliottia-*type strains, as well as the less related *Enterobacter*-type strains (Fig. 1). Whole-genome sequence-based average nucleotide identity (ANI) values among the ten isolates were found to range from 97.68 to 99.9%. Similarly, the in silico DDH (dDDH) values among these isolates ranged from 80.5 to 100%. Furthermore, in a previous study [11], RAPD fingerprinting of these strains were obtained and reported, and these showed that all the strains exhibited over 90% similarity in RAPD profiles (Suppl. Figure 2). This data suggested that the ten isolates belong to the same species but comprised different strains. Furthermore, comparison of 16S rRNA, MLSA, ANI, and dDDH analyses confirmed that all the ten isolates belonged to the same species, consistent with the previous proposal [25]. The ANI and dDDH values between the novel species candidate V104_15^T^ (= LMG 32996^ T^, = DSM 115585^ T^) and previously described *Lelliottia*-type strains were below 92% and 44%, respectively. Additionally, the other 9 isolates from this study showed that the highest ANI value of 91.32% and the highest dDDH value of 43.3% when compared with the closely related type strain *L. jeotgali* PFL01^T^. This indicated that V104_15^T^ and nine isolates represent a novel species, as both the ANI and dDDH analyses values when compared to the other *Lelliottia-*type strains were below the species level cut-off values [25]. In addition, ANI and dDDH values between the novel species candidate V104_15^T^ (= LMG 32996^ T^, = DSM 115585^ T^) strain and other nine isolates from lettuce indicated that these were the same at the species level but not at the subspecies level, according to a previous study that defined minimum standards for the use of genome data, stating that strains of the same subspecies display < 80% and > 70% sequence similarity in dDDH analyses [24] (Suppl. Table 1).

**Fig. 2:**
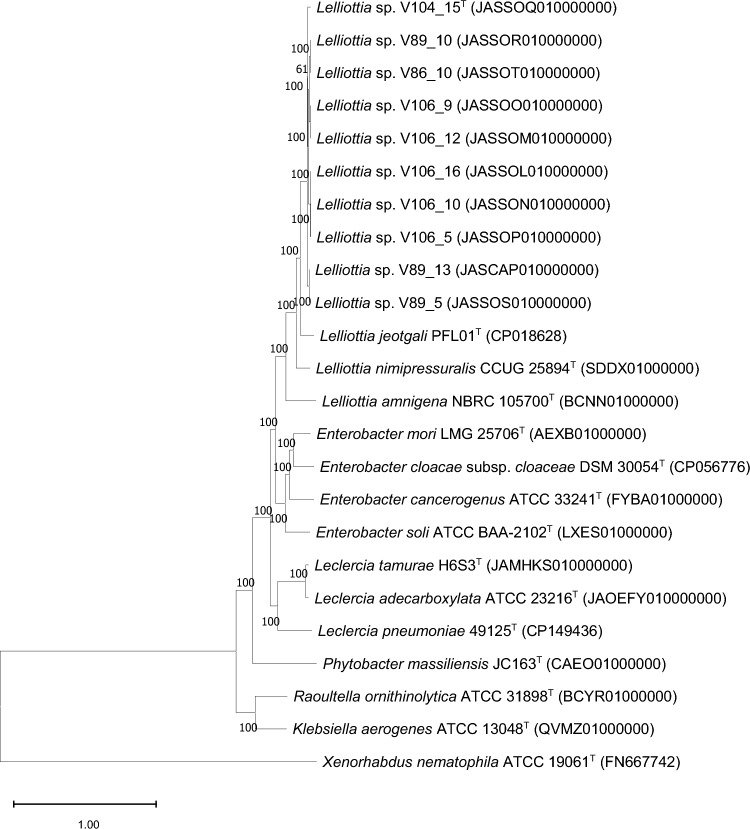
Core genome-based phylogenetic tree reconstructed together with closely related *Lelliottia*-type strains using the RAxML method implanted in BV-BRC server. Fast Bootstrap values were calculated based on 100 replications, with values over 50% being indicated. Bar indicates substitutions per nucleotide position

For phylogenomic analysis, a phylogenetic tree based on whole-genome sequences was reconstructed with closely related type strains using the BV-BRC sever [12]. For this analysis, 500 conserved coding sequences were extracted and concatenated. Subsequently, a homologous group filtering and group alignment were performed. An estimated phylogenetic tree based on the concatenated alignment sequences was calculated using a RAxML pipeline with default parameters. Genome sequences of all our isolates, including the novel species candidate-type strain V104_15^T^ (= LMG 32996^ T^, = DSM 115585^ T^), other *Lelliottia-*type strains (i.e., *L. jeotgali* PFL01^T^ [5], *L. nimipressuralis* CCUG 25894^ T^ [4], and *L. amnigena* NBRC 105700^ T^), and closely related *Enterobacter*, *Klebsiella,* and *Raoultella* strains, were core genome aligned using the mafft pipeline. Rapid bootstrap analysis was then performed using the RAxML pipeline [16] with default parameters. In the phylogenetic trees, all of our isolates, including the novel species candidate V104_15^T^, clustered closely together with other *Lelliottia-*type strains and were again confirmed to belong to the genus *Lelliottia* (Fig. 2). In addition, the V104_15^T^ strain and the other nine isolates grouped separately in a unique branch and were thus clearly delineated from the other type strains of the *Lelliottia* genus (Fig. 2). Type strain *L. jeotgali* PFL01^T^ was the closest *Lelliottia-*type strain to V104_15^T^ (= LMG 32996^ T^, = DSM 115585^ T^) in the phylogenetic analysis. However, the ANI and in silico DDH values between our ten isolates and *L. jeotgali* PFL01^T^ were lower than 92% and 44%, respectively, which suggested that the all strains belong to a single novel species of the genus *Lelliottia* for which the name *Lelliottia wanjuensis* is proposed and for which strain V104_15^T^ is proposed to represent the type strain.

### Cellular Fatty Acid Analysis and Electron Microscopy

**Fig. 3:**
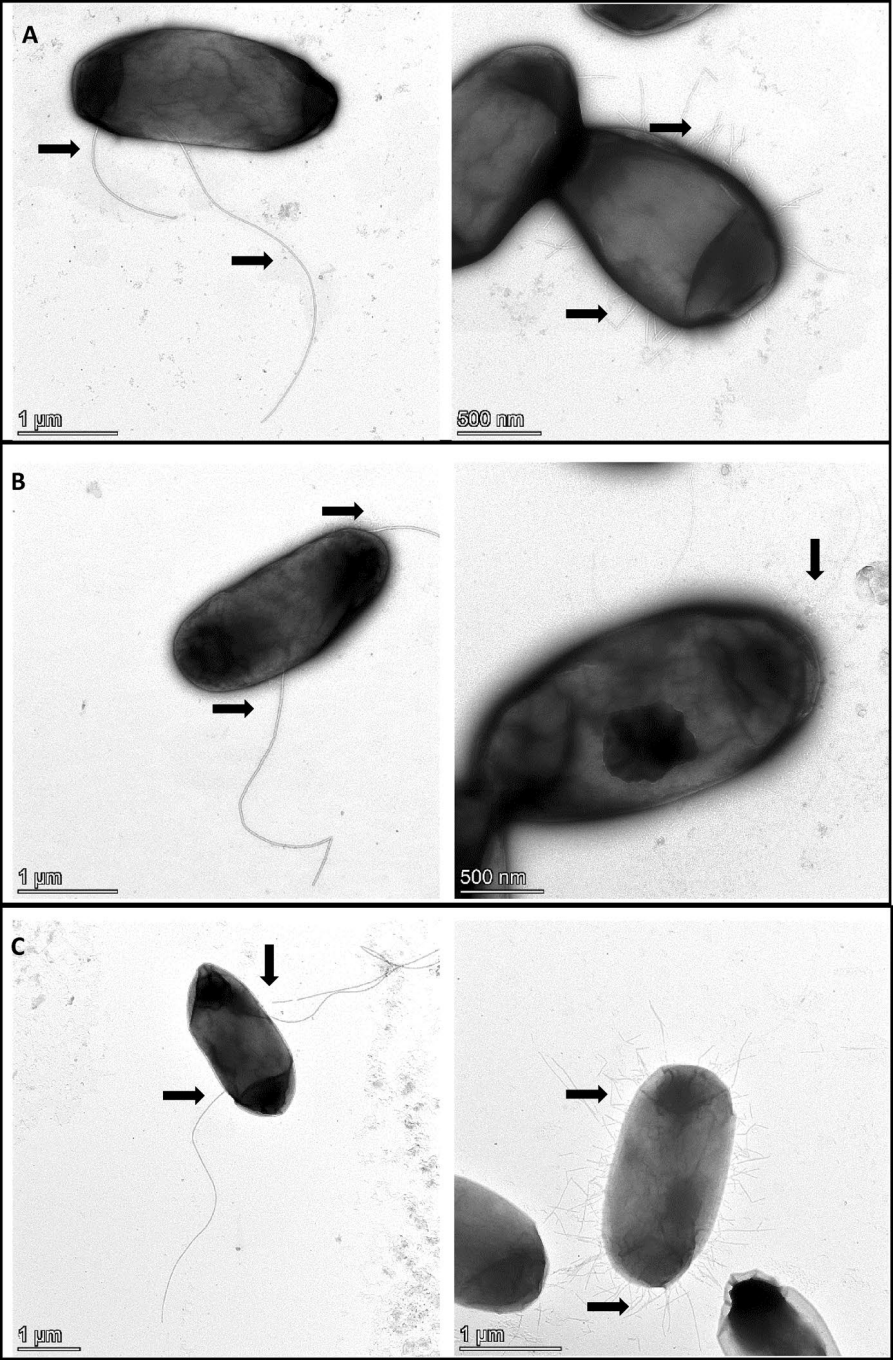
Transmission electron microscopy of *Lelliottia* strains V104_15^T^ (**A**), V86_10 (**B**) and V106_5 (**C**) isolated from lettuce. Cells are straight rod shape and measuring about 2.0– 2.5 µm long with flagellar arrangements. Flagella and pili structures were indicated with arrows

The major cellular fatty acids of strain V104_15^T^ were C_16:0_ (27.7%) and cyclo-C_17:0_ (14%) including summed features (Table 3). The main difference between type strain V104_15^T^ and other *Lelliottia*-type strains was the presence of C_15:0_, and major (over 10%) cellular fatty acids were C_16:0_, cyclo-C_17:0_, summed features 3, and summed features 8 (Table 3). Following the phenotypic assays, the morphological characteristics of all isolates were determined using phase contrast and transmission electron microscopy. Using phase contrast microscopy, all strains appeared as small motile rods. The cell morphologies of representative *L. wanjuensis* strains V104_15^T^, V86_10, and V106_5 are shown in Fig. 3. The cells of these strains showed a straight rod morphology, measuring 1.0 × 2.4 μm for V104_15^T^, 1.1 × 2.1 μm for V86_10, and 1.0 × 2.0 µm for V106_5. Long flagella as well as pili were observed to occur on the cells. These phenotypic and genotypic findings confirmed that the V104_15^T^ strain is a representative strain of a novel species in the genus *Lelliottia* and the name *Lelliottia wanjuensis* sp. nov. is proposed.

**Table 3:** Composition of cellular fatty acids of V104_15^T^ and type strains in the genus *Lelliottia*

Fatty acid	1	2	3	4
Straight-chain saturated				
C_10:0_	–	0.1	–	0.2
C_12:0_	3.3	4.5	3.6	3.7
C_13:0_	0.5	1	1.3	1.3
C_14:0_	5	7.2	6.7	6
C_15:0_	**3.0**	–	–	–
C_16:0_	**27.7**	**27.3**	**27.3**	**23.7**
C_17:0_	2.5	2.7	3.1	2.1
C_18:0_	0.3	–	–	–
Unsaturated				
cyclo-C_17:0_	**14**	**20.4**	**16.9**	4.9
Hydroxy				
C_18:1_ 2-OH	–	0.2	0.3	-
Summed features				
2	7.6	**10**	9.1	**10.6**
3	**15.2**	**12**	**15**	**31.8**
8	**17.8**	**12.9**	**13.9**	**10.9**

The composition of cellular fatty acids of other *Lelliottia*-type strains were obtained from a previous study [5]. Strain: 1, V104_15^T^; 2, *L. jeotgali* PFL01^T^; 3, *L. nimipressuralis* LMG 10245^T^; and 4, *L. amnigena* LMG 2784^T^. Major fatty acids (> 10.0%) and specific fatty acid of V104_15^T^ are denoted in bold. Summed feature 2 (C_16:1_ iso I /C_14:0_ 3-OH), Summed feature 3 (C_16:1_ *ω7c* / C_16:1_ *ω6c*), and Summed feature 8 (C_18:1_ *ω7c* or C_18:1_ *ω6c*)

### Description of *Lelliottia wanjuensis*

*Lelliottia wanjuensis* (Wan.Ju.en’sis. N.L. Fem. Adj. *wanjuensis,* pertaining to Wanju, a city located in South Korea, where the bacterium was first isolated. Cells are Gram stain-negative, facultative anaerobic, oxidase negative, catalase positive, motile, and rod shaped. Growth occurs at 0.5–7% NaCl and at 7, 10, 35, 37, and 39 °C, but not at 41 °C. Colonies on LB medium are round, irregular, slightly raised or slightly convex, gray–white, slightly opaque, and 2–3.5 mm in diameter after 24 h of incubation at 35 °C. Ornithine decarboxylase, arginine dihydrolase, phenol red, *β*-glucosidase, *N*-acetyl-*β*-glucosaminidase, *β*-galactosidase, and α-galactosidase are present, while lysine decarboxylase, urease, lipase, *L*-aspartate-arylamidase, and α-maltosidase are absent. The presence of α-glucosidase and the production of indole from tryptophan are variable. Acid is produced from galacturonate, mannitol, maltose, palatinose, malonate, glucose, sucrose, *L*-arabinose, trehalose, rhamnose, and cellobiose, but not from *L*-arabitol, 5 ketogluconate, adonitol, D-arabitol, inositol, and sorbitol. The major fatty acids are C_16:0_ and cyclo-C_17:0_, including summed feature 8 (C_18:1_ *ω7c* or C_18:1_ *ω6c*). The genome is 4.8–5.3 Mbp in size, with genomic DNA G + C contents of 55.22 to 55.84 mol%.

The type strain is V104_15^T^ (= LMG 32996^ T^, = DSM 115585^ T^). The accession number of the 16S rRNA gene and the genome sequence of the type strain V104_15^T^ are OR946368 and JASSOQ010000000, respectively.

## Supplementary Information

Below is the link to the electronic supplementary material.

Supplementary file1 (DOCX 374 KB)

## Data Availability

The accession number of the 16S rRNA gene and the genome sequence of the type strain V104_15^T^ are OR946368 and JASSOQ010000000, respectively. The accession numbers at DDBJ/ENA/GenBank for the 16S rRNA gene sequences and the draft genomes of *Lelliottia* strains are listed in supplementary Fig. 1 and Table 2, respectively. The type strain V104_15^T^ is accessible from the Belgian Coordinated Collection of microorganisms (BCCM/LMG, = LMG 32996^ T^) and the Deutsche Sammlung von Mikroorganismen und Zellkulturen GmbH (= DSM 115585^ T^).
