# Supplementary material for: Lelliottia wanjuensis sp. nov. Isolated from Korean Lettuce in Wanju, South Korea
Source: Curr Microbiol. 2024 Sep 28;81(11):382. doi: 10.1007/s00284-024-03911-6 (PMC11438706; doi:10.1007/s00284-024-03911-6)
Supplement: Supplementary file 1 — Supplementary file1 (DOCX 374 KB) [file 284_2024_3911_MOESM1_ESM.docx]

|  | *In silico* DDH (%) | | | | | | | | | | |
| --- | --- | --- | --- | --- | --- | --- | --- | --- | --- | --- | --- |
| ANI value (%) | Strain | V86_10 | V89_5 | V89_10 | V89_13^*^ | V104_15^T^ | V106_5 | V106_9 | V106_10 | V106_12 | V106_16 |
|  | V86_10 | - | 97.78% | 97.73% | 97.76% | 97.67% | 97.88% | 98.68% | 97.89% | 98.67% | 97.83% |
|  | V89_5 | 80.8% | - | 99.99% | 99.99% | 97.90% | 97.89% | 97.77% | 97.91% | 97.78% | 97.91% |
|  | V89_10 | 80.8% | 100% | - | 99.99% | 97.89% | 97.88% | 97.75% | 97.85% | 97.73% | 97.87% |
|  | V89_13^*^ | 80.8% | 100% | 100% | - | 97.89% | 97.92% | 97.80% | 97.91% | 97.79% | 97.91% |
|  | V104_15^T^ | 80.6% | 80.9% | 80.9% | 80.9% | - | 97.68% | 97.71% | 97.67% | 97.75% | 97.74% |
|  | V106_5 | 81.9% | 81.6% | 81.6% | 81.6% | 80.8% | - | 97.89% | 99.99% | 97.87% | 99.97% |
|  | V106_9 | 88.8% | 80.5% | 80.5% | 80.5% | 80.9% | 82.5% | - | 97.89% | 99.9% | 97.86% |
|  | V106_10 | 81.9% | 81.6% | 81.6% | 81.6% | 80.8% | 100% | 82.5% | - | 97.87% | 99.97% |
|  | V106_12 | 88.8% | 80.5% | 80.5% | 80.6% | 81.0% | 82.4% | 99.9% | 82.4% | - | 97.88% |
|  | V106_16 | 81.8% | 81.6% | 81.6% | 81.6% | 80.8% | 99.9% | 82.3% | 99.9% | 82.3% | - |

Supplementary Table 1. Average nucleotide identity (ANI) and *in silico* DNA-DNA hybridization values among the *Lelliottia* isolates in this study. ^*^The genome sequence data of V89_13 strain was adapted from previous study [1].

Supplementary Table 2. Phenotypic characteristics of all isolates belonging to the novel species *L. wanjuensis.* +: positive; w: weak positive; -: negative;

| **Characteristics** | **V86_10** | **V89_5** | **V89_10** | **V89_13** | **V106_5** | **V106_9** | **V106_10** | **V106_12** | **V106_16** |
| --- | --- | --- | --- | --- | --- | --- | --- | --- | --- |
| Growth at 41°C | - | - | - | - | - | - | - | - | - |
| Growth at 7°C | + | + | + | + | + | + | + | + | + |
| Growth at 6% (w/v) NaCl | + | + | + | + | + | + | + | + | + |
| Growth at 7% (w/v) NaCl | - | - | - | - | - | - | - | + | - |
| Enzyme activity |  |  |  |  |  |  |  |  |  |
| Ornithine decarboxylase | + | + | + | + | + | + | + | + | + |
| Urease | - | - | - | - | - | - | - | - | - |
| ß-glucosidase | + | + | + | + | + | + | + | + | + |
| ß-galactosidase | + | + | + | + | + | + | + | + | + |
| α-glucosidase | - | - | - | + | w | + | - | + | + |
| α-galactosidase | + | + | + | + | + | + | + | + | + |
| Acid production from |  |  |  |  |  |  |  |  |  |
| D-trehalose | + | + | + | + | + | + | + | + | + |
| L-rhamnose | + | + | + | + | + | + | + | + | + |
| Inositol | - | - | - | - | - | - | - | - | - |
| D-cellobiose | + | + | + | + | + | + | + | + | + |
| D-sorbitol | - | - | - | - | - | - | - | - | - |
| 5 ketogluconate | - | - | - | - | - | - | - | - | - |
| D-mannitol | + | + | + | + | + | + | + | + | + |
| D-maltose | + | + | + | + | + | + | + | + | + |
| Indole (L-Tryptophan) | - | - | - | - | - | - | - | - | - |
| D-glucose | + | + | + | + | + | + | + | + | + |
| D-sucrose | + | + | + | + | + | + | + | + | + |
| L-arabinose | + | + | + | + | + | + | + | + | + |

Supplementary Figure legends

Supple. Fig. 1 Phylogeny of 16S rRNA gene sequences of ten isolates with closely related *Lelliottia* type strains including two closely related *Enterobacter* type strains using the MEGA 11 program. The maximum-likelihood method and Kimura two-parameter model were adapted and bootstrap values were calculated based on 1000 replications. The DDBJ/ENA/GenBank accession numbers for the 16S rRNA gene sequences were indicated.

Suppl. Fig. 2 The fingerprint profile obtained from previous study [1] by UPGMA clustering using the Pearson correlation coefficient of RAPD PCR of 10 *Lelliottia* isolates in this study.

Supplementary Figure 1.

Supplementary Figure 2.

100

95

**RAPD**


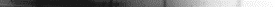

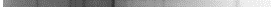

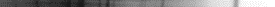

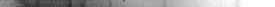

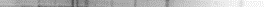

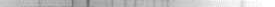

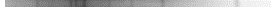

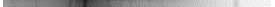

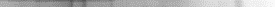

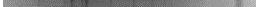


V89_5

V106_16

V89_10

V89_13

V106_5

V106_9

V106_10

V106_12

V104_15^T^

V86_10

1. **Jeong S, Kim I, Kim B-E, Jeong M-I, Oh K-K et al.** Identification and Characterization of Antibiotic-Resistant, Gram-Negative Bacteria Isolated from Korean Fresh Produce and Agricultural Environment. *Microorganisms* 2023;11(5):1241.
